# Live imaging of altered *period1* expression in the suprachiasmatic nuclei of *Vipr2*^−/−^ mice^1^

**DOI:** 10.1111/j.1471-4159.2008.05520.x

**Published:** 2008-08

**Authors:** Alun T L Hughes, Clare Guilding, Laura Lennox, Rayna E Samuels, Douglas G McMahon, Hugh D Piggins

**Affiliations:** * Faculty of Life Sciences, University of ManchesterManchester, UK; † Department of Biological Sciences, Vanderbilt UniversityNashville, Tennessee, USA

**Keywords:** circadian, green fluorescent protein, imaging, *period1*, suprachiasmatic nucleus, vasoactive intestinal polypeptide

## Abstract

Vasoactive intestinal polypeptide and its receptor, VPAC_2_, play important roles in the functioning of the brain’s circadian clock in the suprachiasmatic nuclei (SCN). Mice lacking VPAC_2_ receptors *(Vipr2*^−/−^) show altered circadian rhythms in locomotor behavior, neuronal firing rate, and clock gene expression, however, the nature of molecular oscillations in individual cells is unclear. Here, we used real-time confocal imaging of a destabilized green fluorescent protein (GFP) reporter to track the expression of the core clock gene *Per1* in live SCN-containing brain slices from wild-type (WT) and *Vipr2*^−/−^ mice. Rhythms in *Per1*-driven GFP were detected in WT and *Vipr2*^−/−^ cells, though a significantly lower number and proportion of cells in *Vipr2*^−/−^ slices expressed detectable rhythms. Further, *Vipr2*^−/−^ cells expressed significantly lower amplitude oscillations than WT cells. Within each slice, the phases of WT cells were synchronized whereas cells in *Vipr2*^−/−^ slices were poorly synchronized. Most GFP-expressing cells, from both genotypes, expressed neither vasopressin nor vasoactive intestinal polypeptide. Pharmacological blockade of VPAC_2_ receptors in WT SCN slices partially mimicked the *Vipr2*^−/−^ phenotype. These data demonstrate that intercellular communication via the VPAC_2_ receptor is important for SCN neurons to sustain robust, synchronous oscillations in clock gene expression.

Endogenous near-24 h (circadian) rhythms in brain, behavior, and physiology are generated by cell autonomous circadian oscillators present in neural and non-neural tissues (Herzog 2007). The molecular basis for these daily cellular timekeepers are well established and include the period (*Per1–3*), cryptochrome 1–2, brain and muscle Arnt-like protein-1 genes, and their protein products (Panda *et al.* 2002; Piggins 2002; Reppert and Weaver 2002; Hastings and Herzog 2004; Ko and Takahashi 2006). In the mammalian brain, the dominant circadian clock is localized to the suprachiasmatic nuclei (SCN) of the hypothalamus (Rusak and Zucker 1979; Weaver 1998). Studies of long-term recordings from fetal and neonatal rodent SCN cells dispersed and cultured onto multielectrode plates reveal that these neurons maintain circadian rhythms in spontaneous discharge activity (Welsh *et al.* 1995) with the daily period of these rhythms determined by genotype (Liu *et al.* 1997). Intriguingly, SCN neurons under these conditions can be desynchronized, with electrical activity of adjacent neurons peaking several hours out of phase (Herzog *et al.* 1997 and see Brown and Piggins 2007 for review). Moreover, in SCN cultures from mice bearing a construct in which luciferase (luc) is driven by the mouse *Per1* promoter (*Per1*::luc mice), treatment with tetrodotoxin (which blocks voltage-gated fast sodium currents as well as spontaneous action potential discharge) desynchronizes bioluminescence rhythms of SCN neurons (Yamaguchi *et al.* 2003). Such findings implicate extracellular communication mechanisms in the appropriate phasing of cellular timekeepers in the SCN. Indeed, under certain circumstances, oscillator network interactions in the SCN have recently been demonstrated to compensate for *Per1* or cryptochrome 1 deficiency, maintaining rhythmicity in mutant SCN slices *in vitro* and gross animal behavior *in vivo* (Liu *et al.* 2007).

From mice to humans, a key neurochemical marker of the SCN is vasoactive intestinal polypeptide (VIP) (Piggins and Cutler 2003; Hofman and Swaab 2006; Vosko *et al.* 2007). In rodents, VIP-containing neurons delineate the ventral SCN, where retinal innervation is most dense (Antle and Silver 2005; Morin and Allen 2006). VIP neurons have extensive intra-SCN projections and innervate neurons immunoreactive (-ir) for arginine vasopressin (AVP) in the medial SCN, somatostatin-ir cells in the dorsal SCN, as well as gastrin-releasing peptide-ir neurons of the central SCN, and other VIP neurons (Abrahamson and Moore 2001). The SCN target of VIP, the VPAC_2_ receptor, is heavily expressed in all regions/divisions of the SCN, indicating VIP–VPAC_2_ signaling to be influential in intercellular signaling between SCN neurons (Kalamatianos *et al.* 2004; Kallo *et al.* 2004).

Pharmacological studies have supported this view and further indicate a role in resetting and synchronizing SCN neuronal clock activity. *In vivo*, microinjection of VIP into the SCN region resets rodent behavioral rhythms (Piggins *et al.* 1995), while *in vitro* applications of exogenous VIP to SCN brain slices shifts rhythms in neurophysiological activity (Reed *et al.* 2001) and AVP release (Watanabe *et al.* 2000). Subsequent development of transgenic mice lacking VPAC_2_ expression (*Vipr2*^−/−^ mice; Harmar *et al.* 2002) or deficient in VIP (VIP/PHI^−/−^ mice; Colwell *et al.* 2003) have established that chronic loss of VIP–VPAC_2_ signaling results in grossly disrupted molecular and neurophysiological rhythms in the SCN (Harmar *et al.* 2002; Cutler *et al.* 2003; Hughes *et al.* 2004; Brown *et al.* 2005, 2007) that are accompanied by aberrant wheel-running and metabolic rhythms (Harmar *et al.* 2002; Colwell *et al.* 2003; Hughes *et al.* 2004; Aton *et al.* 2005; Brown *et al.* 2005; Bechtold *et al.* 2008).

Discontinuous assessment of adult *Vipr2*^−/−^ SCN through *in situ* hybridization has revealed abnormally low expression profiles of core clock genes, such as *Per1*, throughout this structure at all circadian timepoints examined, suggesting the complete absence of rhythms in cell autonomous circadian oscillators. However, continuous neurophysiological recordings from adult *Vipr2*^−/−^ SCN brain slices indicate that some neurons sustain low amplitude rhythms (Brown *et al.* 2005). Here, we use confocal microscopy to determine rhythms in a destabilized green fluorescent protein (d2EGFP) construct driven by the *Per1* promoter, *Per1*::d2EGFP (*Per1*::GFP), in living SCN brain slices from adult wild-type (WT) and *Vipr2*^−/−^ mice, and show that *Per1* rhythms are widespread in the adult SCN of both genotypes.

## Materials and methods

### Animals

For this study, locally generated *Vipr2*^−/−^ mice (Harmar *et al.* 2002) crossed with *mPer1*::d2EGFP (*Per1*::GFP; Kuhlman *et al.* 2000) expressing mice were produced. All mice used in this study expressed the *Per1*::GFP transgene and were either homozygous for the disrupted *Vipr2*^−/−^ transgene (*Vipr2*^−/−^ × *Per1*::GFP; referred to as *Vipr2*^−/−^) or homozygous for the WT, non-disrupted, *Vipr2* gene (*Vipr2^+/+^* × *Per1*::GFP strain bred from congenic littermates of original *Vipr2*^−/−^ × *Per1*::GFP stock; referred to as WT). Male and female mice aged at least 6 weeks of age were used for behavioral assessment and young adult mice (mean age: 33 days old) were used for *Per1*::GFP fluorescence imaging. All mice were group housed under a 12 h light : 12 h dark (LD) cycle with *ad libitum* access to food (Beekay, B&K Universal, Hull, UK) and water prior to experimentation. Temperature was maintained at ∼18°C and humidity at ∼40%. All procedures were carried out in accordance with the UK Animals (Scientific Procedures) Act 1986.

### Behavioral Assessment of *Vipr2*^−/−^ and WT mice expressing *Per1*::GFP

To assess wheel-running behavior in LD and dark : dark cycles (DD), WT (*n* = 4) and *Vipr2*^−/−^ (*n* = 15) mice expressing the *Per1*::GFP transgene were individually housed in running wheel-equipped cages (wheel diameter 180 mm) under a 12 h : 12 h LD cycle for 14 days (∼60 lux lights on at cage floor level) then released into DD (0 lux) for at least 12 days. Analysis of locomotor activity under LD conditions was carried out on the final 10 days of LD and under DD conditions on the first 12 days immediately following the LD cycle. Wheel revolutions were recorded in 10 min time bins by the acquisition program DataQuest III (Data Sciences Inc., St Paul, MN, USA), data were then transferred to the Tau program (Mini-Mitter Co., Sunriver, OR, USA) and output to analysis software; TauActo 1.2 (developed in-house by Dr T. Brown) or El Temps (Dr A. Díez-Noguera, Barcelona, Spain). The following ranges of behavioral parameters were analyzed: phase of activity on release into DD; period of the rhythm in DD; and strength of the rhythm (percentage of variance explained by the dominant periodic component of the rhythm; %V) in DD. Unpaired *t*-tests (*p* < 0.05) were used to determine statistically significant genotype differences.

### Culture preparation

Mice were culled by cervical dislocation following halothane (Concord Pharmaceuticals, Essex UK) anesthesia shortly after the time of lights on [Zeitgeber time (ZT) 1-3] and slice cultures prepared under dimmed lights. Following removal, brains were cooled and moistened with ice-cold Hank’s balanced salt solution [Sigma, Poole, UK; supplemented with 0.035% sodium bicarbonate (Sigma), 0.01 M HEPES (Sigma) and 1000 μg/mL penicillin–streptomycin (Gibco Invitrogen Ltd, Paisley, UK)]; 300-μm thick coronal brain slices containing the mid-SCN were cut using a vibroslicer (Camden Instruments, Leicester, UK) and manually trimmed to a small block measuring ∼4 mm wide. Excised tissue was cultured on interface-style Millicell culture inserts [PICMORG50; Millipore (UK) Ltd., Watford, UK] in glass coverslip-based culture dishes (Fluorodish; World Precision Instruments Ltd., Stevenage, UK) containing 1.2 ml of sterile culture medium [DMEM; Dulbecco’s modified Eagle’s medium, D-2902 (Sigma); supplemented with 3.5 g/L d-glucose (Sigma); 0.035% sodium bicarbonate (Sigma); 0.01 M HEPES buffer (Sigma); 1000 μg/mL penicillin–streptomycin (Gibco Invitrogen Ltd), and 5% fetal bovine serum (Gibco Invitrogen Ltd) in autoclaved Milli-Q water]. For VPAC_2_ antagonist treated slices culture medium was further supplemented with 10 nM PG 99-465 (Dr Patrick Robberecht, University of Brussels, Brussels, Belgium). Dishes were sealed with a glass coverslip using autoclaved high-vacuum grease (Dow Corning Ltd., Coventry, UK) and stored in darkness at 37°C for 4–6 h before imaging.

### Confocal imaging of *Per1*::GFP fluorescence in live SCN slice cultures

*Per1*::GFP fluorescence was imaged with either a Leica SP2 or SP5 acousto-optical beam splitter confocal microscope with a 10×/0.3 numerical aperture Fluotar objective (Leica, Milton Keynes, UK) using the 488 nm laser line for excitation and detecting emitted fluorescence between 498 and 525 nm. One 16 image ‘Z’ stack was acquired every hour for the duration of recording, using 3× Kalman averaging to reduce background noise for each individual image. Images of transmitted light were also recorded for each timepoint. Fluorescence images were transferred to ImageJ software (version 1.37a; NIH, Bethesda, MA, USA) and 7 of the 16 ‘Z’ images at each timepoint collapsed to an average projection for analysis. The specific ‘Z’ levels chosen at each timepoint were adjusted as appropriate to correct for drift of the slice in the ‘Z’ plane over time. Relative optical density of individual cells was measured over time using a region of interest tool. All cells visible for at least 48 h were analyzed from slices imaged on the SP2 system; 30 such cells were selected at random for analysis from slices imaged on the SP5 system. Raw fluorescence data were corrected for variations in background brightness by subtracting the optical density value of a standardized, non-GFP expressing, non-SCN region from each data value; corrected data were then smoothed using a 5 h running mean before peak and average fluorescence over the imaging sequence were measured.

Processed fluorescence data were assessed using Clockwise curve fitting software (developed in house by Dr T. Brown) as previously described (Bechtold *et al.* 2008) to determine the period, phase, and significance of circadian variation in *Per1*::GFP fluorescence of individual cells. Briefly, data were normalized such that they spanned a range of values between 100 and −100. Normalized data were fit with the equation *Y* = *A* sin[*B*(*x* + *C*)] using the Newton–Raphson iterative method, where *A* equaled the amplitude of the rhythm, *B* equaled the period in radians/h, and *C* determined the phase. Initial values of *A*, *B*, and *C* were estimated from the best fitting curve of a series of > 3000 standard curves with periodicities between 3 and 34 h and a range of different amplitudes and phasing. Significant rhythmic variation in the data was assessed by repeating the curve fitting procedure 1000 times using the same dataset, but with the order of observations randomized with respect to time. Amplitude (peak-trough 24–48 h after commencement of imaging) was assessed manually. For a proportion of cells, rhythmicity, period, and phase measurements were subsequently confirmed by manual assessment of *Per1*::GFP expression profiles and in all cases found to be in close agreement with Clockwise. SD of the time of peak fluorescence between individual cells within a slice was taken as a measure of synchrony and confirmed using Rayleigh plots (software provided by Dr T. Brown/El Temps). Significant differences between groups were assessed using unpaired *t*-test and anova as appropriate with *p* < 0.05 required for significance.

Preliminary analyses revealed no significant differences within genotypes for period, synchrony, or percentage of rhythmic cells for data acquired using the SP2 and SP5 systems so for analysis of such characteristics, the datasets from the two systems were combined. This, however, would be inappropriate for average fluorescence, peak fluorescence and amplitude data, and though trends in SP2 imaged slices were similar, these are presented for SP5 imaged slices only. Slices imaged and used for analysis were as follows: SP2 WT *n* = 8, SP2 *Vipr2*^−/−^*n* = 7; SP5 WT *n* = 8, SP5 WT treated with 10 nM PG 99-465 *n* = 7, and SP5 *Vipr2*^−/−^*n* = 4.

To assess the spatial distribution of *Per1*::GFP expressing cells within WT and *Vipr2*^−/−^ SCN, time-lapse ‘snapshot’ images for each slice were analyzed and all visible *Per1*::GFP cells assigned a grid reference marking both its medial–lateral and ventral–dorsal location, each on a scale of 1–6 (1 signifying most medial and most ventral and 6 indicating lateral and dorsal locations). The relative abundance of cells in the different medial–lateral and ventral–dorsal areas of the SCN for WT and *Vipr2*^−/−^ slices were assessed using two way anova with *a priori*, single degree of freedom, pairwise comparisons. Medial–lateral and ventral–dorsal sub-SCN location was assessed for correlation with the time of peak fluorescence of cells rhythmically expressing *Per1*::GFP. For this analysis, the SCN was divided into only two regions for each analysis (medial vs. lateral and ventral vs. dorsal).

### Immunohistochemistry

*Per1*::GFP expressing WT and *Vipr2*^−/−^ mice were culled at ZT9 for anti-AVP and anti-VIP immunofluorescence (*n* = 2 each) or across the circadian cycle for anti-GFP nickel di-aminobenzine immunohistochemistry. Brains were processed using standard techniques (see Supplementary material for details). Immunofluorescence and endogenous GFP were imaged using the SP2 confocal microscope and nickel di-aminobenzine-labeled GFP visualized on a light microscope and counted manually.

## Results

### Locomotor activity of *Per1*::GFP expressing WT and *Vipr2*^−/−^ mice

**Fig. 1:**
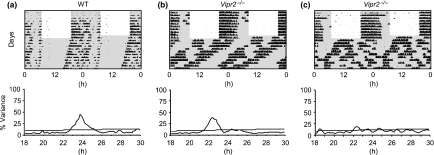
Actograms and periodograms for wheel-running activity of individual WT (a) and *Vipr2*^−/−^ (b and c) mice expressing the *Per1*::GFP transgene. Both WT and *Vipr2*^−/−^ mice synchronize locomotor activity to a 12 h : 12 h LD cycle. All WT mice free-run in constant darkness with a clear, coherent circadian rhythm (a) but *Vipr2*^−/−^ mice express a continuum of behavioral phenotypes from clearly rhythmic (b) to arrhythmic (c). Actograms are double plotted showing two consecutive days data on each line, light and shaded areas represent lights on and off, respectively. Straight diagonal line on periodogram indicates significance at *p* < 0.001.

The generation of *Per1*::GFP expressing WT and *Vipr2*^−/−^ mouse strains had no obvious deleterious effects on the locomotor wheel running activity of these mice (*n* = 4 and *n* = 15, respectively); behavioral profiles were similar to previous descriptions of non-*Per1*::GFP expressing mice for both genotypes (Pittendrigh and Daan 1976; Schwartz and Zimmerman 1990; Hughes *et al.* 2004; Aton *et al.* 2005). Further, *Per1*::GFP expressing WT mouse behavior was similar to that of the original *Per1*::GFP mouse strain (LeSauter *et al.* 2003; Quintero *et al.* 2003). Briefly, WT mice (expressing the *Per1*::GFP transgene) entrained to a 12 h : 12 h LD cycle and free-ran robustly in DD with a mean period and rhythm strength (%V) of 23.63 ± 0.04 h and 34.8 ± 5.8%, respectively (Fig. 1a). *Vipr2*^−/−^ mice (expressing the *Per1*::GFP transgene) synchronized activity to the LD cycle and, as is common for this genotype, commenced locomotor activity shortly after release into DD (10.89 ± 0.75 h prior to the phase of activity under LD; Fig. 1b and c). In DD, the behavioral phenotypes of *Vipr2*^−/−^ mice defined a continuum from robustly rhythmic with a single dominant component of locomotor behavior (Fig. 1b) to apparent arrhythmicity with multiple, low power periodic components (Fig. 1c). Those *Vipr2*^−/−^ individuals expressing a clear, dominant rhythmic component to their locomotor activity (∼50%) did so with a mean period of 22.50 ± 0.12 h, significantly shorter than that of their WT counterparts (*p* < 0.0001). Further, the strength of the dominant rhythmic component for *Vipr2*^−/−^ mice, when present, was significantly lower than observed for WT mice (%V 22.5 ± 3.8% vs. 34.8 ± 5.8%, respectively; *p* < 0.05).

### Confocal imaging of *Per1*::GFP expression in live SCN containing brain slices *in vitro*

**Fig. 2:**
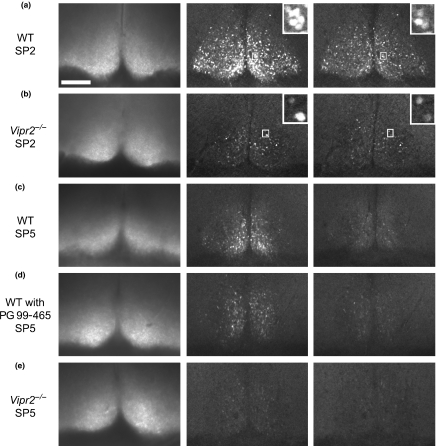
Transmitted light and *Per1*::GFP fluorescence time-lapse confocal photomicrograph images of the SCN from WT mice (a and c) and *Vipr2*^−/−^ mice (b and e). Panel (d) shows the SCN of a WT slice maintained with 10 nM PG 99-465 in the culture medium. Slices shown in panels (a and b) were imaged on the SP2 microscope system while panels (c–e) show slices imaged on the SP5 microscope system. Fluorescence images presented were taken 12 h apart between 24 and 48 h in culture. Note bright fluorescence signal and clear peak-trough differences for untreated WT SCN (a and c). *Vipr2*^−/−^ SCN contained fewer detectable *Per1*::GFP expressing cells with lower levels of fluorescence and less distinct peak-trough differences *en masse*. Inset windows for panel (a) show higher magnification of a cluster of individual WT cells oscillating in phase, while inset window for panel (b) shows two *Vipr2*^−/−^ cells oscillating out of phase. Scale bar, 150 μm.

Longitudinal confocal imaging of SCN containing brain slices from both WT and *Vipr2*^−/−^ mice yielded images of sufficient quality and resolution such that individual cells within each slice could be detected and analyzed (see Fig. 2 and Movies S1–S5). In total 1290 cells from WT slices were analyzed, 596 cells from *Vipr2*^−/−^ slices and 210 cells from WT slices treated with PG 99-465.

**Fig. 3:**
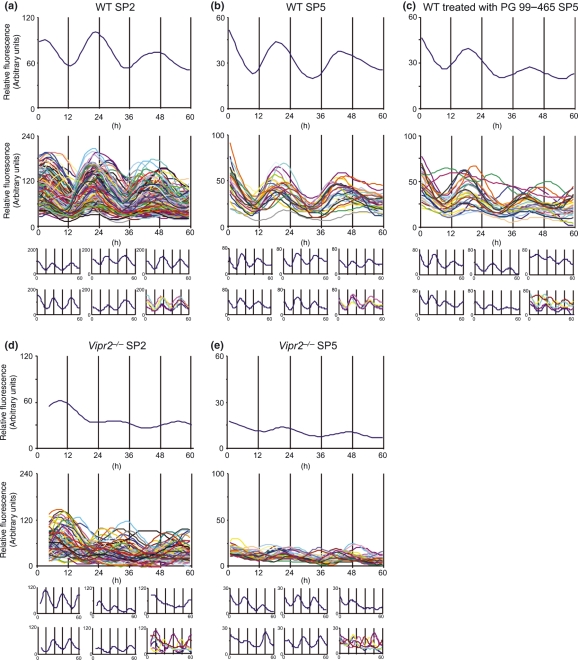
Confocal *Per1*::GFP fluorescence traces from the SCN of WT mice (a and b) and *Vipr2*^−/−^ mice (d and e). Panel (c) shows traces from the SCN of a WT slice maintained with 10 nM PG 99-465 in the culture medium. Upper trace on each panel shows a multiunit profile generated by averaging the fluorescence of all individual cells analyzed at each timepoint. Middle trace on each panel shows all individual cells detectable (a and d) or the 30 analyzed cells selected at random (b, c, and e). Lower six mini-traces show a selection of fluorescence profiles for individual cells to highlight their rhythmicity and these five cells plotted together to highlight synchrony. Slices shown in panels (a and d) were imaged on the SP2 microscope system and are directly comparable with each other while slices shown in panels (b, c, and e) were imaged on the SP5 microscope system and are directly comparable with one another but not (a and d). Note higher levels of fluorescence and higher amplitude and synchrony of rhythms in WT slices compared with *Vipr2*^−/−^ (panels a vs. d and b vs. e). WT slices treated with PG 99-465 maintained the level of fluorescence and amplitude observed in untreated WT SCN, however, synchrony of rhythms was partially disrupted (panels c vs. b and e).

All imaged WT slices (*n* = 16) expressed overt rhythms in *Per1*-driven GFP expression that were clearly distinguishable on visual inspection of time-lapse images (e.g. Fig. 2a and c and Movies S1 and S3) and multiunit rhythm profiles (Fig. 3a and b). Ten of 11 imaged *Vipr2*^−/−^ SCN slices displayed readily identifiable circadian variation in *Per1*-driven GFP [e.g. Figs 2b and 3(d and e) and Movies S2 and S5]. The remaining *Vipr2*^−/−^ slice appeared, on initial visual inspection of time-lapse confocal imagery to express no clear circadian rhythm in GFP expression (Fig. 2e), however, closer scrutiny of its multiunit expression profile revealed a very low amplitude rhythmic expression pattern (not shown). In contrast, discontinuous sampling of *Vipr2*^−/−^ mice with assessment of *Per1*::GFP expression using immunohistochemistry, failed to reveal circadian expression of GFP-immunoreactivity (Fig. S1). Immunohistochemical assessment of WT *Per1*::GFP expression revealed clear circadian variation (Fig. S1).

Using confocal imaging of live slices, on average, 131 *Per1*::GFP-expressing SCN cells were detected in each WT slice and 68 cells per slice from *Vipr2*^−/−^ mice. Within WT slices, 89.7 ± 2.4% of imaged cells expressed a statistically significant circadian rhythm in *Per1*::GFP fluorescence as determined by Clockwise analysis. In *Vipr2*^−/−^ slices, however, the percentage of rhythmic *Per1*::GFP cells was significantly reduced; 59.1 ± 6.8% of cells expressing a significant rhythm (*p* < 0.0001 vs. WT).

Rhythmic cells in WT slices expressed *Per1*::GFP fluorescence with a mean period of 23.69 ± 0.17 h, similar to the period of free-running locomotor activity of these mice in DD (23.63 ± 0.04 h). The mean period of GFP fluorescence expressed by *Vipr2*^−/−^ cells (23.45 ± 0.11 h) did not, however, closely match that observed for locomotor behavior of rhythmic individuals of this genotype (22.50 ± 0.12 h) and was not significantly different to the mean period of WT cells (*p* > 0.05).

**Fig. 4:**
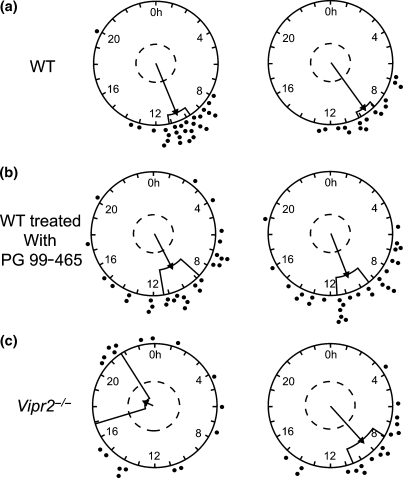
Example Rayleigh plots showing phase clustering of *Per1*::GFP fluorescence profiles for rhythmic cells from WT (a), *Vipr2*^−/−^ (c), and WT cells treated with PG 99-465 (b). *Per1*::GFP expressing cells in all WT SCN (*n* = 16) were significantly phase clustered (mean *p* < 0.0001) whereas only ∼50% of *Vipr2*^−/−^ slices (*n* = 6 of 11) achieved significant phase clustering of cellular rhythms. Those *Vipr2*^−/−^ slices which did reach significance were less significantly clustered than WT slices (mean ‘*r*’ 0.52 ± 0.06 vs. 0.80 ± 0.02 for WT slices). WT SCN treated with PG 99-465 all displayed significant phase clustering of rhythmic fluorescence profiles, but with ‘*r*’ values intermediate to untreated WT and *Vipr2*^−/−^ slices (mean ‘*r*’ 0.73 ± 0.02). Filled circles indicate the phase of individual cells. Straight line indicates the mean phase vector with the surrounding box indicating the variance of phase of individual cells while the inner broken line indicates the significance threshold of *p* = 0.05.

The two genotypes differed in the mean phase at which the GFP fluorescence signal of individual cells peaked; cells in *Vipr2*^−/−^ slices phase lagged WT cells by ∼2 h; mean peak phase for *Vipr2*^−/−^ cells ZT12.29 ± 0.57 h vs. ZT10.03 ±0.30 h for WT cells (*p* < 0.005). Further, the rhythms of *Per1*::GFP fluorescence expressed by cells in *Vipr2*^−/−^ slices were significantly less synchronized than those of WT cells [see Fig. 3(a vs. d) and (b vs. e)]. Taken as an indirect measure of synchrony, analysis of the SD of peak time for all cells within each slice revealed a significantly greater variation in peak time between individual cells (mean SD) for *Vipr2*^−/−^ cells than WT (*p* < 0.0001). This genotype difference in cellular synchrony was confirmed with the use of Rayleigh plots; all WT slices (*n* = 16) expressed significant phase clustering of *Per1*::GFP rhythm phases (all *p* < 0.0001) whereas only 6 of 11 *Vipr2*^−/−^ slices expressed significantly phase clustered cellular rhythms (*p* < 0.005–0.0001 for significant *Vipr2*^−/−^ Rayleigh plots). ‘*r*’ values returned from Rayleigh tests (the length of the Rayleigh vector; a measure inversely proportional to variability within the dataset) indicated that even for significantly phase clustered *Vipr2*^−/−^ rhythms, synchrony was significantly reduced with respect to WT rhythms (mean significant *Vipr2*^−/−^‘*r*’: 0.52 ± 0.06 vs. 0.80 ± 0.02 for WT slices; *p* < 0.0001; see Fig. 4a vs. c).

For the analysis of amplitude, average, and peak fluorescence, only data from slices imaged on the SP5 confocal system were used to ensure the validity of direct comparisons between groups and identical excitation and detection settings. The mean amplitude of GFP fluorescence rhythms expressed by *Vipr2*^−/−^ cells (3.33 ± 0.47, arbitrary units; *n* = 4) was significantly lower than that measured for cells in WT slices (16.60 ± 2.27; *p* < 0.00; *n* = 8; Fig. 3b vs. e). Similarly, comparison of both average and peak fluorescence between WT and *Vipr2*^−/−^ cells revealed significantly lower levels in *Vipr2*^−/−^ tissue [16.28 ± 2.86 vs. 2.58 ± 2.76, arbitrary units (*p* < 0.01) and 26.31 ± 3.76 vs. 4.56 ± 2.21, arbitrary units (*p* < 0.005), respectively; Fig. 3b vs. e]. The direction and significance of these trends in amplitude, average and peak fluorescence of *Per1*::GFP expression were maintained for data collected using the SP2 imaging system (data not shown).

### PG 99-465 partially mimics the *Vipr2*^−/−^ molecular phenotype

All WT slices treated with the VPAC_2_ antagonist, PG 99-465 (*n* = 7; all imaged on the SP5 system), displayed clear circadian variation in *Per1*-driven GFP expression (Figs 2d and 3c and Movie S4). The period of cellular rhythms in *Per1*::GFP following PG 99-465 treatment was significantly reduced compared with both WT and *Vipr2*^−/−^ cells (22.83 ± 0.25; *p* < 0.01 and *p* < 0.05, respectively, vs. WT and *Vipr2*^−/−^). PG 99-465 did not, however, alter the percentage of cells detected that expressed a significant circadian rhythm (89.4 ± 4.9%; *p* > 0.05 vs. WT; *p* < 0.01 vs. *Vipr2*^−/−^), the amplitude of rhythms (14.33 ± 1.50, arbitrary units) or average/peak levels of expression (20.69 ± 1.84/30.25 ± 2.28, arbitrary units) from WT levels (all *p* > 0.05 vs. WT).

Despite the failure of PG 99-465 to mimic the phenotype of *Vipr2*^−/−^ mice for the above characteristics of *Per1*::GFP expression rhythms, cellular synchrony in WT slices treated with PG 99-465 was partially modified towards that of *Vipr2*^−/−^ slices. Mean SD of peak time of individual cells in PG 99-465 treated WT slices was 3.91 ± 0.41; intermediate to the values of 2.27 ± 0.22 and 5.78 ± 0.54 for WT and *Vipr2*^−/−^ cells, respectively [*p* < 0.0005 vs. WT and *p* < 0.05 vs. *Vipr2*^−/−^; Fig. 3(c vs. b and e)]. Rayleigh analysis revealed all PG 99-465 treated WT slices (*n* = 7) to express significant phase clustering of cellular rhythms (*p* < 0.05–< 0.0001). ‘*r*’ values, however, indicated significantly reduced clustering compared with WT slices (*p* < 0.05) and significantly greater phase clustering than for *Vipr2*^−/−^ (*p* < 0.001) slices (mean PG 99-465 treated WT ‘*r*’ = 0.73 ± 0.02 vs. 0.80 ± 0.02 and 0.52 ± 0.06 for untreated WT and *Vipr2*^−/−^ slices, respectively; Fig. 4).

### Spatial and temporal analysis of distribution of *Per1*::GFP expressing cells within the WT and *Vipr2*^−/−^ SCN

**Fig. 5:**
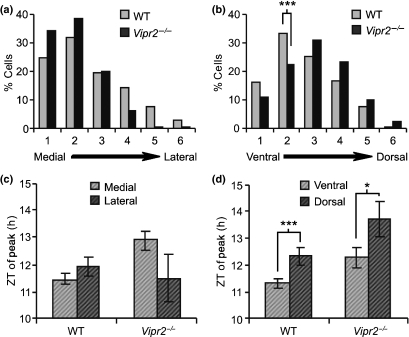
Histograms showing the medial–lateral (a) and ventral–dorsal (b) distribution of rhythmic *Per1*::GFP expressing cells in the SCN of WT and *Vipr2*^−/−^ mice. The medial–lateral distribution of rhythmic cells showed no significant difference between genotypes whereas in the WT SCN, rhythmic cells were located significantly more ventrally than in the *Vipr2*^−/−^ SCN. No significant difference was seen for either genotype in the phase at which cells reached peak fluorescence between the medial and lateral halves of the SCN (c), however, cells in the ventral SCN peaked significantly earlier than those in the dorsal SCN in both WT and *Vipr2*^−/−^ mice (d); *p < 0.05 and ***p < 0.005.

Analysis of the location of *Per1*::GFP expressing cells within WT and *Vipr2*^−/−^ SCN revealed a tendency for significantly rhythmic cells to be located along the medial border, with no significant difference in the medial–lateral distribution of cells between the two genotypes (*p* > 0.05; Fig. 2 and 5a). Ventral–dorsal sub-SCN location of rhythmic *Per1*::GFP cells did, however, differ between genotypes; in WT slices, cells were most frequently located just dorsal to the ventral boundary (ventral–dorsal region 2), whereas in *Vipr2*^−/−^ slices cells were most commonly located ventral–dorsally in the mid-SCN region (ventral–dorsal region 3; *p* < 0.05 between genotypes; Fig. 5b). Indeed, ventral–dorsal region 2 contained a significantly higher proportion of rhythmic cells in WT slices than *Vipr2*^−/−^ (*p* < 0.005; Fig. 5b). Cells expressing *Per1*::GFP arrhythmically were located with the same medial–lateral distribution as rhythmic cells, though ventral–dorsally, arrhythmic cells were dispersed more evenly throughout the SCN (data not shown).

The sub-SCN location of *Per1*::GFP cells expressing a significant circadian variation was correlated with the time of their peak fluorescence. No significant differences in the time of peak *Per1*::GFP fluorescence were found between the medial and lateral SCN for either genotype (both *p* > 0.05; Fig. 5c) though in both WT and *Vipr2*^−/−^ slices cells peaked significantly earlier in the ventral area of the SCN than the dorsal (*p* < 0.005 and *p* < 0.05, respectively; Fig. 5d).

### Co-expression of arginine vasopressin and vasoactive intestinal polypeptide with *Per1*::GFP

**Fig. 6:**
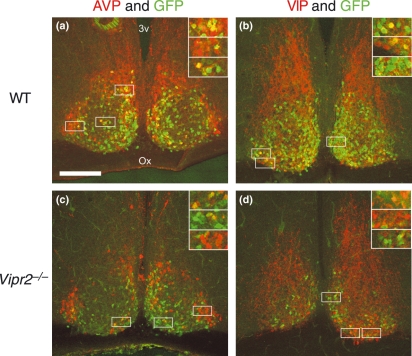
Confocal photomicrographs showing *Per1*::GFP fluorescence (green) and either AVP (a and c) or VIP (b and d) immunoreactivity (red) in fixed SCN tissue from WT (a and b) and *Vipr2*^−/−^ (c and d) mice. Note co-localization of *Per1*::GFP with both AVP and VIP in both genotypes but that the majority of *Per1*::GFP-expressing cells do not express either AVP or VIP. Insets show magnified images of areas marked by boxes. Scale bar, 150 μm.

To gain some insight into the neurochemical phenotype of *Per1*::GFP expressing cells and assess potential deficiency of *Per1*::GFP expression in the *Vipr2*^−/−^ SCN in two substantial cell populations, fixed SCN-containing *Per1*::GFP expressing brain slices of both genotypes were immunolabeled for AVP and VIP. While *Per1*::GFP expressing cells were found to co-localize with both AVP and VIP cells in the SCN of both genotypes (Fig. 6), qualitative assessment based on the small sample assessed here found the majority of *Per1*::GFP expressing cells to co-localize with neither neuropeptide. The majority of VIP-ir cells in both genotypes also expressed *Per1*::GFP (Fig. 6b and d), however, AVP-ir cells were commonly observed to only be single-labeled (Fig. 6a and c), with only a minority also expressing *Per1*::GFP.

## Discussion

The results of this study extend our knowledge of the capacity for neuronal timekeeping in mice with deficient neuropeptide signaling. Consistent with earlier reports (Quintero *et al.* 2003; Maywood *et al.* 2006), we found that the vast majority (∼90%) of *Per1*::GFP expressing SCN neurons from WT mice generate circadian rhythms in *Per1*-driven fluorescence. All WT SCN slices (16/16) showed significant clustering of time of peak fluorescence between individual cells, whereas a reduced proportion of *Vipr2*^−/−^ slices did so, reflecting the fraction (roughly half) of *Vipr2*^−/−^ mice lacking detectable behavioral rhythms. Importantly, our finding that a surprisingly large proportion (∼59%) of *Vipr2^−/−^Per1*::GFP expressing SCN neurons generate detectable fluorescence rhythms when maintained *in vitro* (albeit with reduced amplitude and synchrony) demonstrates that discontinuous sampling protocols [clock gene mRNA (Harmar *et al.* 2002) and clock gene reporter protein (Fig. S1 of this study), c-Fos and phosphorylated extra-cellular signal-regulated kinase protein (Hughes *et al.* 2004), and electrophysiological activity (Cutler *et al.* 2003)] are not sufficiently sensitive to accurately determine circadian pacemaking capacity in animals with low amplitude SCN activity. Indeed, our observation of the absence of synchrony in *Per1*::GFP expression in the *Vipr2*^−/−^ SCN reveals the limitation of discontinuous assessment approaches. Rhythms in *Per1*::GFP were not unique to any one subcompartment of either the WT or *Vipr2*^−/−^ SCN, although a greater proportion of these cells were present in the medial SCN in both. More rhythmic cells were seen in ventral regions of the WT SCN than elsewhere, whereas in the *Vipr2*^−/−^ SCN most cells were located at central levels of the dorsoventral axis. Our immunohistochemical analysis indicates that the neurochemical phenotypes of *Per1*::GFP expressing cells includes VIP-ir and AVP-ir cells in the SCN of both genotypes though the majority of *Per1*::GFP cells appear to express neither VIP-ir nor AVP-ir. We also reveal for the first time that transient treatment of WT SCN with PG 99-465 disrupted the synchrony, but not the proportion of cells rhythmically expressing *Per1*::GFP or the amplitude of such molecular rhythms.

### Behavioral screening of *Per1*::GFP expressing mice

The circadian wheel-running rhythms of (*Per1*::GFP expressing) WT mice in this study (period ∼23.6 h) are broadly similar to those reported earlier using this mouse (∼23.5 h; Kuhlman *et al.* 2000). Moreover, this is also close to the period of C57BL6 mice which lack the *Per1*::GFP construct (23.77 h, Schwartz and Zimmerman 1990; 23.7–23.8 h, Hughes *et al.* 2004; Hughes and Piggins 2008). The range of circadian behavioral phenotypes seen in *Per1*::GFP expressing *Vipr2*^−/−^ mice (∼50% lacking clear rhythms and ∼50% expressing short period rhythms of ∼22.5 h) is similar to previous descriptions of the wheel-running rhythms of non-*Per1*::GFP expressing *Vipr2*^−/−^ mice (Hughes *et al.* 2004; Aton *et al.* 2005; Brown *et al.* 2005; Hughes and Piggins 2008). Therefore, on neither WT nor *Vipr2*^−/−^ backgrounds does this construct appear to grossly alter the period of murine circadian locomotor rhythms.

### *Per1*::GFP expression in the WT and *Vipr2*^−/−^ SCN

Wild-type tissue generated robust, near 24 h, rhythmic oscillations in *Per1*::GFP expression when averaged for all detectable cells (multiunit rhythm). This produced a global SCN rhythm with a period closely matching that of locomotor behavior (Schwartz and Zimmerman 1990; Kuhlman *et al.* 2000). These findings are consistent with previous descriptions of global SCN *Per1* expression in live mouse tissue *in vitro*, reported by both *Per1*::GFP (using the same construct as the present work; Quintero *et al.* 2003) and *Per1*-luc (Yamaguchi *et al.* 2000, 2003). In the present study, ∼90% of imaged WT cells expressed a detectable circadian rhythm in *Per1*::GFP fluorescence. This percentage is similar to that reported for the original stock of *Per1*::GFP mice (89% in slices from animals housed in LD and 74% from slices taken from animals housed in DD; Quintero *et al.* 2003).

Observations from the present investigation complement previous reports of continuously monitored electrophysiological activity (Aton *et al.* 2005; Brown *et al.* 2005) and *Per* gene driven reporter construct expression in WT and *Vipr2*^−/−^ mice (Quintero *et al.* 2003; Maywood *et al.* 2006). The large sample size we examined (1290 WT and 596 *Vipr2^−/−^Per1*::GFP SCN cells) augments and extends on the study of Maywood *et al.* (2006) which focused mostly on neurochemical manipulation *Per1*::luc expression in WT and *Vipr2*^−/−^ mice (and did not assess behavior). Here, we reveal that a notable subset (∼59%) of imaged *Vipr2*^−/−^ SCN cells rhythmically express *Per1*-driven GFP in a circadian, and often robust, fashion. These rhythms are of lower amplitude and are expressed by a significantly smaller proportion of cells than in the WT SCN, but are, on average, of similar periodicity. *In vivo* we detected clear genotype differences in the period of wheel-running and the absence of such a difference in SCN period *in vitro* could be due to culture conditions or may reflect disruption in SCN control of behavior in the *Vipr2*^−/−^ mouse (and see below). Both Aton *et al.* (2005) and Brown *et al.* (2005) observed the same trend in electrical activity as is reported here for *Per1*::GFP fluorescence.

The estimated synchrony between *Per1*-driven GFP-expressing cells in the *Vipr2*^−/−^ SCN in our study was greatly altered compared with WT mice; this is in general agreement with Aton *et al.* (2005) and Maywood *et al.* (2006) who reported similar desynchrony of electrical activity and *Per1*::luc bioluminescence, respectively, between rhythmic SCN neurons of *Vipr2*^−/−^ mice. Transient blockade of VPAC_2_ receptors in WT SCN with PG 99-465 disrupted the estimated synchrony but not the amplitude of the rhythm, suggesting that the well-documented suppressive effects of the *Vipr2*^−/−^ transgene on the amplitude of *Per1* expression and SCN electrophysiological activity arise through longer term impairments in VIP–VPAC_2_ receptor signaling.

The oscillations generated by individual *Vipr2*^−/−^ cells showed significant phase clustering in a reduced number of slices compared with those in WT slices (6 of 11; ∼55% vs. 16 of 16; 100%); where significant phase clustering was observed, the variability between phases expressed within a slices was greater than in WT slices (mean ‘*r*’ 0.52 ± 0.06 vs. 0.80 ± 0.02 for WT) and showed a greater dispersion (see variance boxes on Rayleigh plots; Fig. 4). The reduced peak-trough amplitude of *Per1*::GFP fluorescence detected here, together with lower average and peak fluorescence levels expressed in the *Vipr2*^−/−^ SCN are indicative of perturbation of clock function, an observation mirrored in the reduced circadian amplitude (power; %V) of wheel-running behavior reported for rhythmic *Vipr2*^−/−^ mice (Fig. 1; Aton *et al.* 2005; Hughes and Piggins 2008) as well as the tendency of *Vipr2*^−/−^ SCN neurons to be hyperpolarized when compared with WT SCN neurons (Pakhotin *et al.* 2006).

In the present study, we were able to visualize a greater number of *Per1*::GFP expressing cells in WT slices than in *Vipr2*^−/−^ slices. Supported by the higher levels of expression in WT cells compared with the *Vipr2*^−/−^ SCN, this demonstrates that, overall, WT SCN are possessed of a greater drive towards *Per1* transcription than *Vipr2*^−/−^ SCN. As both Brown *et al.* (2005) and Aton *et al.* (2005) report no difference in average SCN neuronal firing rates between WT and *Vipr2*^−/−^ slices, this difference in molecular expression is apparently not translated to electrical output. Alternatively, these differences raise the possibility that *Per1*::GFP positive and negative neurons in the SCN are fundamentally distinct in their electrical activity across the circadian cycle. Indeed, the absence of overt genotype differences in the period of SCN *Per1*::GFP expression observed here is in contrast to that seen with electrophysiological recordings (Brown *et al.* 2005).

The spatial distribution of *Per1*::GFP expressing cells visualized in this study differed between WT and *Vipr2*^−/−^ mice. WT cells were located predominantly in the medial and ventral SCN, while *Vipr2*^−/−^ cells, though still predominantly medial, were most commonly located at mid-levels of the dorsoventral plane, defining a significantly different neurochemical architecture between the SCN of WT mice and those lacking VIP–VPAC_2_ signaling. Analysis of the time of peak fluorescence of individually rhythmic cells versus their spatial location revealed no significant medial–lateral trend in the SCN of either genotype, however, in both genotypes, *Per1*::GFP expressing cells in the ventral SCN peaked earlier than those of the more dorsal areas of the SCN. Quintero *et al.* (2003), from their own investigation of WT SCN slices, report a slightly different result to that presented here; cells peaked earlier laterally than medially in this study. The authors do not, however, comment on ventral–dorsal distribution of peak phases. The association of ventral location with early phase, described here, is likely a contributory factor towards the earlier mean peak phase of *Per1*::GFP in WT SCN cells compared with *Vipr2*^−/−^. Together, these data suggest heterogeneity in the effects of the loss of VIP–VPAC_2_ signaling on cells in different locations within the SCN. Our observation of ventral SCN cells peaking earlier than dorsal is in close agreement with the relative phases of peak neuronal firing in the ventral and dorsal SCN of WT mice assessed using extracellular recording (Dr T. Brown, unpublished observations).

Based on our immunohistochemistry data, while some AVP-ir and VIP-ir neurons of both genotypes appear to express *Per1*::GFP, the majority of *Per1*::GFP-expressing neurons appear not to express either AVP-ir or VIP-ir. Thus, while the distribution of *Per1*::GFP expressing cells in both genotypes generally matches that of AVP-ir and VPAC_2_ expression (Abrahamson and Moore 2001; Kallo *et al.* 2004; Morin and Allen 2006), contrary to previous interpretation (Maywood *et al.* 2006), the wider neurochemical phenotype of these cells remains unknown. It remains a possibility, however, that AVP and/or VIP are expressed in a greater percentage of *Per1*::GFP cells though at levels which are not detectable with the experimental protocols employed here.

### Treatment of WT slices with PG 99-465

PG 99-465 treatment of WT slices results in a partial mimic of *Vipr2*^−/−^ phenotype. Amplitude and magnitude of fluorescence are not altered, and similarly, percentage of cells rhythmic is not altered by PG 99-465 treatment. However, synchrony of rhythmic cells is partially reduced to levels intermediate to those of WT and *Vipr2*^−/−^ slices. In previous work, we were able to completely duplicate the *Vipr2*^−/−^ phenotype in SCN neuronal firing using chronic perfusion of PG 99-465 to WT SCN slices (Cutler *et al.* 2003; Brown *et al.* 2005). The failure here to replicate this result is most likely due to PG 99-465 treatment in the present study acting as an acute treatment. The single dose added to culture medium at set up is likely broken down in this experimental system such that the VPAC_2_ receptors are not pharmacologically blocked throughout fluorescence recording. This removal from culture of the antagonist as an active compound would equate to an acute rather than chronic treatment, thus resulting in the partial desynchrony of *Per1*::GFP expressing cells observed, without phenocopying other *Vipr2*^−/−^ characteristics.

## Summary and conclusions

In summary, the data presented here show that neurons of the *Vipr2*^−/−^ SCN are capable of producing circadian variation, though a smaller proportion do so than in the WT SCN, and rhythms that are produced by individual cells are less robust. In the SCN of mice lacking VIP–VPAC_2_ signaling, synchrony of *Per1* driven rhythms between those neurons which remain rhythmic is significantly reduced. This demonstrates that VIP–VPAC_2_ signaling in the SCN functions in both rhythm generation and coordination. A role in cell–cell communication signaling from the VIP-ergic to other areas of the SCN appears to be crucial to the generation of high amplitude, synchronized oscillations by neurons of this region and subsequently impacts upon the generation of rhythms.

## Abbreviations used

%V: percentage of variance
AVP: arginine vasopressin
d2EGFP: destabilized green fluorescent protein
DD: dark : dark cycle
GFP: green fluorescent protein
ir: immunoreactive
LD: light : dark cycle
luc: luciferase
*Per1*: *period1*
SCN: suprachiasmatic nuclei
VIP: vasoactive intestinal polypeptide
WT: wild-type
ZT: Zeitgeber time

## Supplementary material

The following supplementary material is available for this article:

**Fig. S1** Histogram showing GFP-immunoreactive (-ir) cell counts in the SCN of WT and *Vipr2*^−/−^ mice across the circadian cycle.

**Movie S1** WT SCN slice imaged over ∼60 h on SP2 confocal microscope.

**Movie S2** *Vipr2*^−/−^ SCN slice imaged over ∼60 h on SP2 confocal microscope.

**Movie S3** WT SCN slice imaged over ∼60 h on SP5 confocal microscope.

**Movie S4** WT SCN slice treated with PG 99-465 imaged over ∼60 h on SP5 confocal microscope.

**Movie S5** *Vipr2*^−/−^ SCN slice imaged over ∼60 h on SP5 confocal microscope.

This material is available as part of the online article from: http://www.blackwell-synergy.com/doi/abs/10.1111/j.1471-4159.2008.05220.x. (This link will take you to the article abstract).
