# Supplementary figures and images for: Live imaging of altered period1 expression in the suprachiasmatic nuclei of Vipr2−/− mice
Source: J Neurochem. 2008 Aug;106(4):1646–57. doi: 10.1111/j.1471-4159.2008.05520.x (PMC2658715; doi:10.1111/j.1471-4159.2008.05520.x)

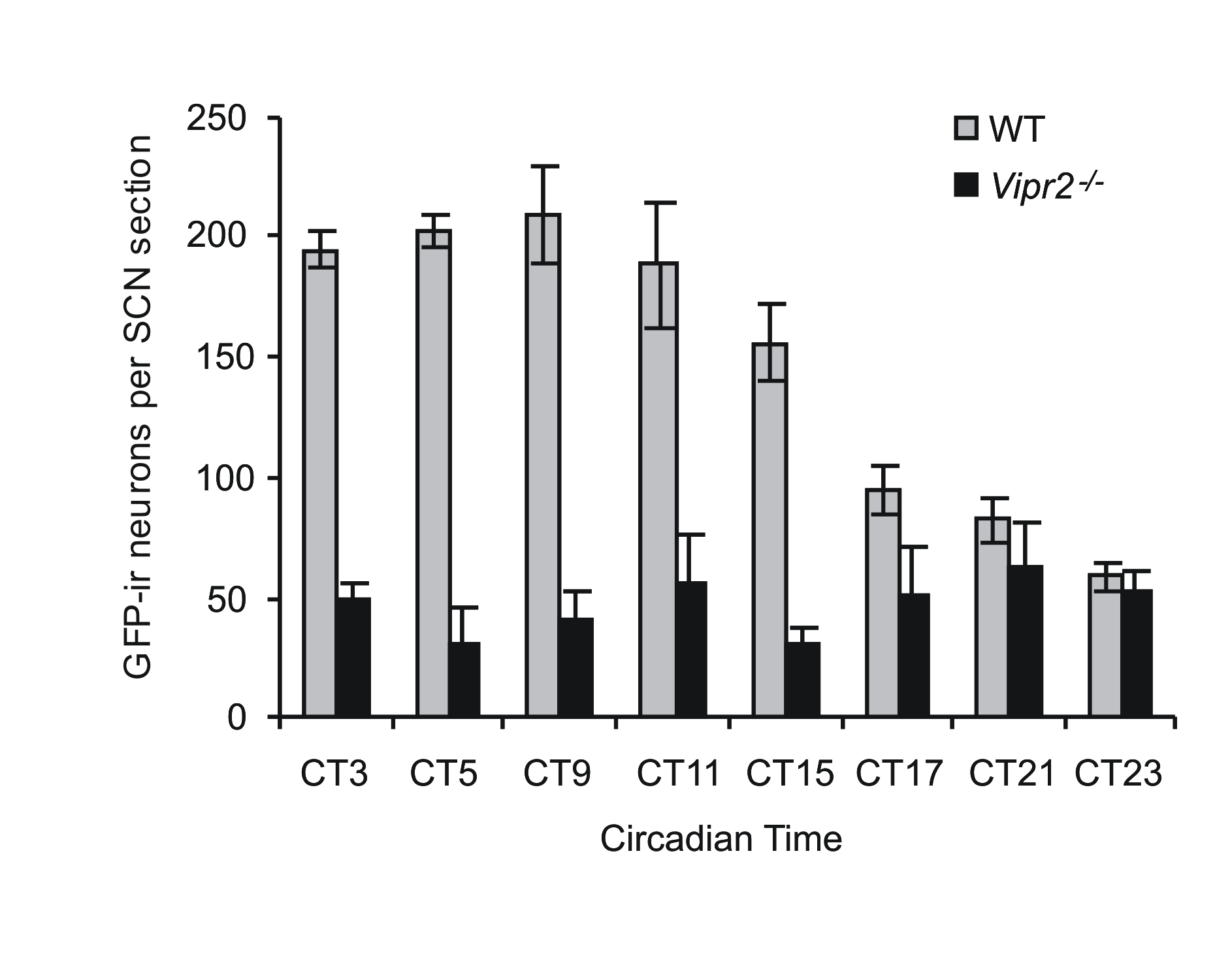

Supplement: Supplementary file 1 [file jnc0106-1646-SD1.tif]
